# 1-(4-Isopropyl­phen­yl)-5-(4-methoxy­phen­yl)pyrazolidin-3-one

**DOI:** 10.1107/S1600536808009823

**Published:** 2008-04-16

**Authors:** Hong-Sheng Jia, Yu-Feng Li, Yuan-Yuan Liu, Shan Liu, Hong-Jun Zhu

**Affiliations:** a Department of Applied Chemistry, College of Science, Nanjing University of Technology, Nanjing 210009, People’s Republic of China

## Abstract

In the mol­ecule of the title compound, C_19_H_22_N_2_O_2_, the pyrazolidinone ring has an envelope conformation, with the C atom attached to the 4-methoxy­phenyl ring displaced by 0.354 (3) Å from the plane of the other ring atoms. The 4-iso­propyl­phenyl ring is oriented with respect to the 4-meth­oxy­phenyl ring at a dihedral angle of 88.94 (3)°. Intra­molecular C—H⋯N hydrogen bonds result in the formation of two planar five-membered rings, which are oriented with respect to the adjacent 4-isopropyl­phenyl and 4-meth­oxy­phenyl rings at dihedral angles of 4.05 (3) and 0.50 (3)°, respectively. In the crystal structure, inter­molecular N—H⋯O hydrogen bonds link the mol­ecules into centrosymmetric dimers.

## Related literature

For general background, see: Menozzi *et al.* (1990 ▶); Brooks *et al.* (1990 ▶); Greenwood *et al.* (1995 ▶).
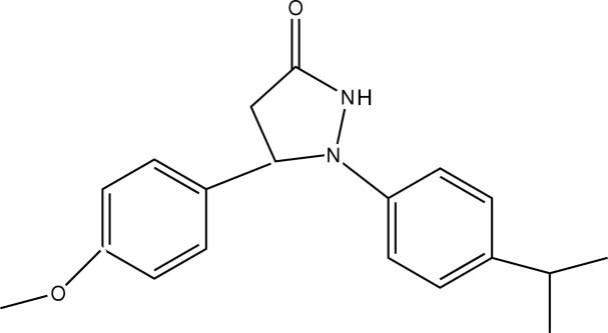


## Experimental

### 

#### Crystal data

C_19_H_22_N_2_O_2_
*M* *_r_* = 310.39
Monoclinic, 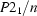

*a* = 14.737 (3) Å
*b* = 7.1490 (14) Å
*c* = 17.493 (4) Å
β = 112.03 (3)°
*V* = 1708.4 (7) Å^3^
*Z* = 4
Mo *K*α radiation
μ = 0.08 mm^−1^
*T* = 294 (2) K
0.40 × 0.30 × 0.20 mm

#### Data collection

Enraf–Nonius CAD-4 diffractometer
Absorption correction: ψ scan (North *et al.*, 1968 ▶) *T*_min_ = 0.969, *T*_max_ = 0.984
3472 measured reflections
3340 independent reflections
1835 reflections with *I* > 2σ(*I*)
*R*_int_ = 0.052
3 standard reflections frequency: 120 min intensity decay: none

#### Refinement

*R*[*F* ^2^ > 2σ(*F* ^2^)] = 0.078
*wR*(*F* ^2^) = 0.163
*S* = 1.06
3340 reflections
202 parameters
1 restraint
H-atom parameters constrained
Δρ_max_ = 0.38 e Å^−3^
Δρ_min_ = −0.78 e Å^−3^

### 

Data collection: *CAD-4 Software* (Enraf–Nonius, 1989 ▶); cell refinement: *CAD-4 Software*; data reduction: *XCAD4* (Harms & Wocadlo, 1995 ▶); program(s) used to solve structure: *SHELXS97* (Sheldrick, 2008 ▶); program(s) used to refine structure: *SHELXL97* (Sheldrick, 2008 ▶); molecular graphics: *SHELXTL* (Sheldrick, 2008 ▶) and *PLATON* (Spek, 2003 ▶); software used to prepare material for publication: *SHELXTL*.

## Supplementary Material

Crystal structure: contains datablocks global, I. DOI: 10.1107/S1600536808009823/hk2451sup1.cif

Structure factors: contains datablocks I. DOI: 10.1107/S1600536808009823/hk2451Isup2.hkl

Additional supplementary materials:  crystallographic information; 3D view; checkCIF report

## Figures and Tables

**Table 1:** Hydrogen-bond geometry (Å, °)

*D*—H⋯*A*	*D*—H	H⋯*A*	*D*⋯*A*	*D*—H⋯*A*
N2—H2*A*⋯O1^i^	0.86	1.96	2.819 (4)	175
C8—H8*A*⋯N2	0.93	2.44	2.761 (5)	100
C14—H14*A*⋯N1	0.93	2.54	2.887 (5)	102

Symmetry code: (i) 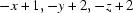.

**Fig. 1.**
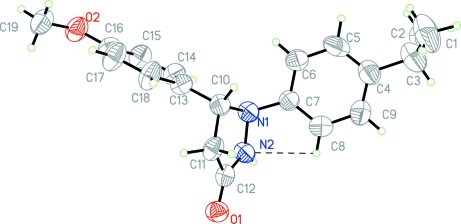
The molecular structure of the title molecule, with the atom-numbering scheme. Displacement ellipsoids are drawn at the 30% probability level. Hydrogen bond is shown as dashed line.

**Fig. 2.**
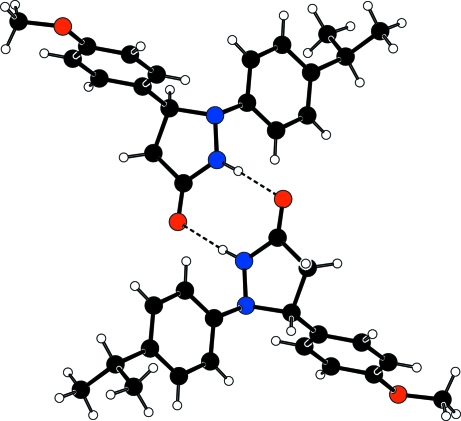
A partial packing diagram of (I). Hydrogen bonds are shown as dashed lines.

## supplementary crystallographic information

### Comment

Pyrazolidin-3-one derivatives are important chemical materials of effective medicines used for treatment of inflammation. They are of great interest because of their biological properties, such as antipyretic activity (Menozzi *et al.*, 1990), liphoxygenase enzyme inhibition (Brooks *et al.*, 1990) and cholecystokinin (CCK) receptor antagonist activity (Greenwood *et al.*, 1995). We report herein the crystal structure of the title compound, (I).

In the molecule of (I), (Fig. 1), rings A (C4-C9) and C (C13-C18) are, of course, planar. The dihedral angle between them is A/C = 88.94 (3)°. Ring B (N1/N2/C10-C12) has envelope conformation with atom C10 displaced by 0.354 (3) Å from the plane of the other ring atoms. The intramolecular C-H···N hydrogen bonds (Table 1) result in the formation of two planar five-membered rings D (N1/N2/C7/C8/H8A) and E (N1/C10/C13/C14/H14A). They are oriented with respect to the adjacent rings at dihedral angles of A/D = 4.05 (3)° and C/E = 0.50 (3)°. So, they are also nearly coplanar.

In the crystal structure, intermolecular N-H···O hydrogen bonds (Table 1) link the molecules into centrosymmetric dimers (Fig. 2), in which they may be effective in the stabilization of the structure.

### Experimental

For the preparation of the title compound, ethanolamine (4 ml) and n-butanol (20 ml) were added to a solution of sodium (40 mmol) in anhydrous methanol (9 mol). Then, the methanol was removed by distillation and 3-(4-methylphenyl) acrylate was added. The mixture was refluxed for 1 h at the temperature above 373 K, after which isopropyphenyl hydrazine (4 ml) was added. The reactants were refluxed for a further 10 h, left to cool to room temperature, and then acidified with acetic acid (36%), allowed to stand, filtered, and the filter cake was crystallized from ethyl acetate to give the title compound (m.p. 435-437 K). It was crystallized by the slow evaporation of an ethyl acetate solution.

### Refinement

H atoms were positioned geometrically, with N-H = 0.86 Å (for NH) and C-H = 0.93, 0.98, 0.97 and 0.96 Å for aromatic, methine, methylene and methyl H, respectively, and constrained to ride on their parent atoms with U_iso_(H) = xU_eq_(C,N), where x = 1.5 for methyl H and x = 1.2 for all other H atoms.

### Crystal data

**Table:**

C_19_H_22_N_2_O_2_	*F*_000_ = 664
*M**_r_* = 310.39	*D*_x_ = 1.207 Mg m^−^^3^
Monoclinic, *P*2_1_/*n*	Mo *K*α radiation λ = 0.71073 Å
Hall symbol: -P 2yn	Cell parameters from 25 reflections
*a* = 14.737 (3) Å	θ = 9–13º
*b* = 7.1490 (14) Å	µ = 0.08 mm^−^^1^
*c* = 17.493 (4) Å	*T* = 294 (2) K
β = 112.03 (3)º	Block, colorless
*V* = 1708.4 (7) Å^3^	0.40 × 0.30 × 0.20 mm
*Z* = 4	

### Data collection

**Table:**

Enraf–Nonius CAD-4 diffractometer	*R*_int_ = 0.052
Radiation source: fine-focus sealed tube	θ_max_ = 26.0º
Monochromator: graphite	θ_min_ = 1.6º
*T* = 294(2) K	*h* = −18→16
ω/2θ scans	*k* = 0→8
Absorption correction: ψ scan(North *et al.*, 1968)	*l* = 0→21
*T*_min_ = 0.969, *T*_max_ = 0.984	3 standard reflections
3472 measured reflections	every 120 min
3340 independent reflections	intensity decay: none
1835 reflections with *I* > 2σ(*I*)	

### Refinement

**Table:**

Refinement on *F*^2^	Secondary atom site location: difference Fourier map
Least-squares matrix: full	Hydrogen site location: inferred from neighbouring sites
*R*[*F*^2^ > 2σ(*F*^2^)] = 0.078	H-atom parameters constrained
*wR*(*F*^2^) = 0.163	*w* = 1/[σ^2^(*F*_o_^2^) + (0.008*P*)^2^ + 3.2*P*] where *P* = (*F*_o_^2^ + 2*F*_c_^2^)/3
*S* = 1.06	(Δ/σ)_max_ < 0.001
3340 reflections	Δρ_max_ = 0.38 e Å^−^^3^
202 parameters	Δρ_min_ = −0.78 e Å^−^^3^
1 restraint	Extinction correction: none
Primary atom site location: structure-invariant direct methods	

### Special details

**Table:**

Geometry. All e.s.d.'s (except the e.s.d. in the dihedral angle between two l.s. planes) are estimated using the full covariance matrix. The cell e.s.d.'s are taken into account individually in the estimation of e.s.d.'s in distances, angles and torsion angles; correlations between e.s.d.'s in cell parameters are only used when they are defined by crystal symmetry. An approximate (isotropic) treatment of cell e.s.d.'s is used for estimating e.s.d.'s involving l.s. planes.
Refinement. Refinement of F^2^ against ALL reflections. The weighted R-factor wR and goodness of fit S are based on F^2^, conventional R-factors R are based on F, with F set to zero for negative F^2^. The threshold expression of F^2^ > 2sigma(F^2^) is used only for calculating R-factors(gt) etc. and is not relevant to the choice of reflections for refinement. R-factors based on F^2^ are statistically about twice as large as those based on F, and R- factors based on ALL data will be even larger.

### Fractional atomic coordinates and isotropic or equivalent isotropic displacement parameters (Å^2^)

**Table:**

	*x*	*y*	*z*	*U*_iso_*/*U*_eq_	
O1	0.52570 (18)	0.7545 (4)	1.04046 (16)	0.0569 (7)	
N1	0.2835 (2)	0.9106 (4)	0.97974 (18)	0.0486 (8)	
N2	0.3849 (2)	0.9266 (4)	0.99532 (18)	0.0508 (8)	
H2A	0.4104	1.0278	0.9859	0.061*	
C1	0.0956 (5)	1.2768 (9)	1.2266 (4)	0.117 (2)	
H1A	0.0772	1.3608	1.2611	0.176*	
H1B	0.1383	1.1821	1.2601	0.176*	
H1C	0.0380	1.2191	1.1876	0.176*	
O2	0.0840 (2)	0.3384 (4)	0.67816 (16)	0.0656 (8)	
C2	0.0897 (4)	1.5470 (7)	1.1350 (3)	0.091	
H2B	0.0705	1.6238	1.1714	0.136*	
H2C	0.0323	1.5035	1.0906	0.136*	
H2D	0.1294	1.6189	1.1131	0.136*	
C3	0.1471 (4)	1.3827 (7)	1.1817 (3)	0.0881 (16)	
H3A	0.2057	1.4352	1.2244	0.106*	
C4	0.1840 (3)	1.2577 (6)	1.1290 (3)	0.0657 (11)	
C5	0.1216 (3)	1.1481 (7)	1.0656 (3)	0.0792 (14)	
H5A	0.0549	1.1507	1.0548	0.095*	
C6	0.1557 (3)	1.0349 (6)	1.0180 (3)	0.0684 (12)	
H6A	0.1116	0.9649	0.9754	0.082*	
C7	0.2539 (3)	1.0251 (5)	1.0332 (2)	0.0467 (9)	
C8	0.3165 (3)	1.1359 (5)	1.0951 (2)	0.0538 (10)	
H8A	0.3831	1.1348	1.1053	0.065*	
C9	0.2817 (3)	1.2487 (6)	1.1424 (3)	0.0626 (11)	
H9A	0.3257	1.3203	1.1843	0.075*	
C10	0.2671 (3)	0.7054 (5)	0.9853 (2)	0.0485 (9)	
H10A	0.2251	0.6878	1.0169	0.058*	
C11	0.3691 (3)	0.6265 (5)	1.0361 (2)	0.0522 (10)	
H11A	0.3780	0.6123	1.0936	0.063*	
H11B	0.3792	0.5064	1.0147	0.063*	
C12	0.4378 (3)	0.7731 (5)	1.0257 (2)	0.0473 (9)	
C13	0.2185 (3)	0.6143 (5)	0.9026 (2)	0.0489 (9)	
C14	0.1919 (3)	0.7046 (6)	0.8282 (2)	0.0556 (10)	
H14A	0.2044	0.8320	0.8274	0.067*	
C15	0.1471 (3)	0.6114 (6)	0.7545 (2)	0.0583 (11)	
H15A	0.1289	0.6768	0.7050	0.070*	
C16	0.1289 (3)	0.4205 (6)	0.7537 (2)	0.0544 (10)	
C17	0.1560 (3)	0.3291 (6)	0.8282 (2)	0.0691 (12)	
H17A	0.1439	0.2017	0.8292	0.083*	
C18	0.2002 (3)	0.4224 (5)	0.9003 (2)	0.0636 (12)	
H18A	0.2188	0.3565	0.9497	0.076*	
C19	0.0726 (3)	0.1427 (6)	0.6765 (3)	0.0721 (13)	
H19A	0.0399	0.1019	0.6206	0.108*	
H19B	0.0344	0.1084	0.7083	0.108*	
H19C	0.1358	0.0845	0.6996	0.108*	

### Atomic displacement parameters (Å^2^)

**Table:**

	*U*^11^	*U*^22^	*U*^33^	*U*^12^	*U*^13^	*U*^23^
O1	0.0495 (16)	0.0490 (16)	0.0681 (17)	0.0004 (13)	0.0174 (13)	0.0023 (13)
N1	0.0495 (18)	0.0428 (17)	0.0569 (19)	−0.0016 (15)	0.0238 (15)	0.0036 (15)
N2	0.0493 (18)	0.0411 (17)	0.068 (2)	−0.0024 (15)	0.0287 (16)	0.0015 (16)
C1	0.158 (6)	0.112 (5)	0.131 (5)	0.002 (4)	0.110 (5)	−0.012 (4)
O2	0.0709 (19)	0.0627 (19)	0.0500 (16)	−0.0031 (15)	0.0077 (14)	−0.0046 (14)
C2	0.091	0.091	0.091	0.000	0.034	0.000
C3	0.098 (4)	0.083 (4)	0.105 (4)	0.008 (3)	0.064 (3)	−0.003 (3)
C4	0.071 (3)	0.059 (3)	0.078 (3)	−0.003 (2)	0.042 (2)	−0.006 (2)
C5	0.051 (2)	0.092 (4)	0.105 (4)	0.001 (3)	0.040 (3)	−0.009 (3)
C6	0.054 (2)	0.068 (3)	0.089 (3)	−0.016 (2)	0.034 (2)	−0.020 (3)
C7	0.047 (2)	0.042 (2)	0.053 (2)	−0.0051 (17)	0.0211 (18)	−0.0005 (18)
C8	0.051 (2)	0.055 (2)	0.055 (2)	−0.0008 (19)	0.0204 (19)	0.011 (2)
C9	0.074 (3)	0.057 (3)	0.061 (3)	−0.007 (2)	0.029 (2)	−0.008 (2)
C10	0.051 (2)	0.044 (2)	0.055 (2)	−0.0040 (18)	0.0254 (18)	−0.0009 (18)
C11	0.063 (2)	0.041 (2)	0.046 (2)	−0.0069 (19)	0.0129 (18)	−0.0012 (17)
C12	0.056 (2)	0.044 (2)	0.042 (2)	−0.0043 (19)	0.0191 (17)	−0.0029 (17)
C13	0.047 (2)	0.045 (2)	0.050 (2)	−0.0016 (18)	0.0125 (17)	0.0050 (18)
C14	0.057 (2)	0.045 (2)	0.058 (2)	−0.0012 (19)	0.014 (2)	0.0083 (19)
C15	0.065 (3)	0.056 (3)	0.046 (2)	0.001 (2)	0.0116 (19)	0.008 (2)
C16	0.045 (2)	0.061 (3)	0.049 (2)	−0.0008 (19)	0.0084 (17)	0.000 (2)
C17	0.089 (3)	0.046 (2)	0.057 (3)	−0.012 (2)	0.010 (2)	0.001 (2)
C18	0.090 (3)	0.042 (2)	0.049 (2)	−0.008 (2)	0.014 (2)	0.0026 (19)
C19	0.075 (3)	0.069 (3)	0.067 (3)	−0.017 (3)	0.020 (2)	−0.017 (2)

### Geometric parameters (Å, °)

**Table:**

O1—C12	1.229 (4)	C7—C8	1.379 (5)
N1—N2	1.418 (4)	C8—C9	1.383 (5)
N1—C7	1.429 (4)	C8—H8A	0.9300
N1—C10	1.496 (4)	C9—H9A	0.9300
N2—C12	1.336 (4)	C10—C13	1.501 (5)
N2—H2A	0.8600	C10—C11	1.538 (5)
C1—C3	1.488 (6)	C10—H10A	0.9800
C1—H1A	0.9600	C11—C12	1.515 (5)
C1—H1B	0.9600	C11—H11A	0.9700
C1—H1C	0.9600	C11—H11B	0.9700
O2—C16	1.368 (4)	C13—C14	1.372 (5)
O2—C19	1.407 (5)	C13—C18	1.396 (5)
C2—C3	1.498 (6)	C14—C15	1.380 (5)
C2—H2B	0.9600	C14—H14A	0.9300
C2—H2C	0.9600	C15—C16	1.390 (5)
C2—H2D	0.9600	C15—H15A	0.9300
C3—C4	1.524 (6)	C16—C17	1.378 (5)
C3—H3A	0.9800	C17—C18	1.358 (5)
C4—C9	1.370 (5)	C17—H17A	0.9300
C4—C5	1.386 (6)	C18—H18A	0.9300
C5—C6	1.384 (6)	C19—H19A	0.9600
C5—H5A	0.9300	C19—H19B	0.9600
C6—C7	1.372 (5)	C19—H19C	0.9600
C6—H6A	0.9300		
			
N2—N1—C7	112.7 (3)	C4—C9—H9A	119.1
N2—N1—C10	104.5 (3)	C8—C9—H9A	119.1
C7—N1—C10	115.0 (3)	N1—C10—C13	113.1 (3)
C12—N2—N1	115.2 (3)	N1—C10—C11	104.5 (3)
C12—N2—H2A	122.4	C13—C10—C11	114.0 (3)
N1—N2—H2A	122.4	N1—C10—H10A	108.3
C3—C1—H1A	109.5	C13—C10—H10A	108.3
C3—C1—H1B	109.5	C11—C10—H10A	108.3
H1A—C1—H1B	109.5	C12—C11—C10	103.3 (3)
C3—C1—H1C	109.5	C12—C11—H11A	111.1
H1A—C1—H1C	109.5	C10—C11—H11A	111.1
H1B—C1—H1C	109.5	C12—C11—H11B	111.1
C16—O2—C19	117.2 (3)	C10—C11—H11B	111.1
C3—C2—H2B	109.5	H11A—C11—H11B	109.1
C3—C2—H2C	109.5	O1—C12—N2	125.8 (3)
H2B—C2—H2C	109.5	O1—C12—C11	126.8 (3)
C3—C2—H2D	109.5	N2—C12—C11	107.4 (3)
H2B—C2—H2D	109.5	C14—C13—C18	116.9 (4)
H2C—C2—H2D	109.5	C14—C13—C10	125.0 (3)
C1—C3—C2	113.1 (4)	C18—C13—C10	118.1 (3)
C1—C3—C4	112.9 (4)	C13—C14—C15	121.7 (4)
C2—C3—C4	112.7 (4)	C13—C14—H14A	119.1
C1—C3—H3A	105.8	C15—C14—H14A	119.1
C2—C3—H3A	105.8	C14—C15—C16	120.4 (4)
C4—C3—H3A	105.8	C14—C15—H15A	119.8
C9—C4—C5	116.7 (4)	C16—C15—H15A	119.8
C9—C4—C3	121.0 (4)	O2—C16—C17	125.1 (4)
C5—C4—C3	122.4 (4)	O2—C16—C15	116.9 (4)
C6—C5—C4	122.0 (4)	C17—C16—C15	118.0 (4)
C6—C5—H5A	119.0	C18—C17—C16	121.0 (4)
C4—C5—H5A	119.0	C18—C17—H17A	119.5
C7—C6—C5	120.5 (4)	C16—C17—H17A	119.5
C7—C6—H6A	119.8	C17—C18—C13	122.0 (4)
C5—C6—H6A	119.8	C17—C18—H18A	119.0
C6—C7—C8	118.0 (4)	C13—C18—H18A	119.0
C6—C7—N1	117.5 (3)	O2—C19—H19A	109.5
C8—C7—N1	124.4 (3)	O2—C19—H19B	109.5
C7—C8—C9	121.0 (4)	H19A—C19—H19B	109.5
C7—C8—H8A	119.5	O2—C19—H19C	109.5
C9—C8—H8A	119.5	H19A—C19—H19C	109.5
C4—C9—C8	121.7 (4)	H19B—C19—H19C	109.5
			
C7—N1—N2—C12	112.7 (3)	C7—N1—C10—C11	−103.1 (3)
C10—N1—N2—C12	−12.9 (4)	N1—C10—C11—C12	−21.7 (4)
C1—C3—C4—C9	−120.1 (5)	C13—C10—C11—C12	102.3 (3)
C2—C3—C4—C9	110.2 (5)	N1—N2—C12—O1	176.9 (3)
C1—C3—C4—C5	59.8 (7)	N1—N2—C12—C11	−1.5 (4)
C2—C3—C4—C5	−69.8 (6)	C10—C11—C12—O1	−163.7 (4)
C9—C4—C5—C6	0.0 (7)	C10—C11—C12—N2	14.7 (4)
C3—C4—C5—C6	−180.0 (4)	N1—C10—C13—C14	0.7 (5)
C4—C5—C6—C7	1.2 (8)	C11—C10—C13—C14	−118.5 (4)
C5—C6—C7—C8	−2.2 (6)	N1—C10—C13—C18	179.5 (4)
C5—C6—C7—N1	−177.8 (4)	C11—C10—C13—C18	60.3 (5)
N2—N1—C7—C6	173.6 (3)	C18—C13—C14—C15	1.4 (6)
C10—N1—C7—C6	−66.8 (5)	C10—C13—C14—C15	−179.8 (4)
N2—N1—C7—C8	−1.8 (5)	C13—C14—C15—C16	−1.0 (6)
C10—N1—C7—C8	117.9 (4)	C19—O2—C16—C17	−5.9 (6)
C6—C7—C8—C9	2.1 (6)	C19—O2—C16—C15	174.9 (4)
N1—C7—C8—C9	177.4 (4)	C14—C15—C16—O2	179.8 (3)
C5—C4—C9—C8	0.0 (7)	C14—C15—C16—C17	0.6 (6)
C3—C4—C9—C8	179.9 (4)	O2—C16—C17—C18	−179.9 (4)
C7—C8—C9—C4	−1.1 (6)	C15—C16—C17—C18	−0.7 (7)
N2—N1—C10—C13	−103.6 (3)	C16—C17—C18—C13	1.2 (7)
C7—N1—C10—C13	132.3 (3)	C14—C13—C18—C17	−1.5 (7)
N2—N1—C10—C11	21.0 (4)	C10—C13—C18—C17	179.6 (4)

### Hydrogen-bond geometry (Å, °)

**Table:**

*D*—H···*A*	*D*—H	H···*A*	*D*···*A*	*D*—H···*A*
N2—H2A···O1^i^	0.86	1.96	2.819 (4)	175
C8—H8A···N2	0.93	2.44	2.761 (5)	100
C14—H14A···N1	0.93	2.54	2.887 (5)	102

Symmetry codes: (i) −*x*+1, −*y*+2, −*z*+2.

## References

[bb1] Brooks, D. W., Basha, A., Gunn, B. P. & Bhatia, P. A. (1990). US Patent No. 4 970 210.

[bb2] Enraf–Nonius (1989). *CAD-4 Software* Enraf–Nonius, Delft, The Netherlands.

[bb3] Greenwood, B., Helton, D. R. & Howbert, J. J. (1995). US Patent No. 5 399 565.

[bb4] Harms, K. & Wocadlo, S. (1995). *XCAD4* University of Marburg, Germany.

[bb5] Menozzi, G., Mosti, L. & Schenone, P. (1990). *Il Farmaco*, **45**, 167–186. 2133993

[bb6] North, A. C. T., Phillips, D. C. & Mathews, F. S. (1968). *Acta Cryst.* A**24**, 351–359.

[bb7] Sheldrick, G. M. (2008). *Acta Cryst.* A**64**, 112–122. 10.1107/S0108767307043930 18156677

[bb8] Spek, A. L. (2003). *J. Appl. Cryst.* **36**, 7–13.

